# Incidence and Risk Factors for Hip Fractures Among U.S. Armed Forces Active Component Women Compared to Men, 2018–2022

**Published:** 2024-08-20

**Authors:** Patricia A. Vu, Shauna L. Stahlman, Michael T. Fan, Natalie Y. Wells

**Affiliations:** 1 Fort Leonard Wood Department of Public Health, MO; 2 Armed Forces Health Surveillance Division, Silver Spring, MD

## Abstract

Women, who comprise approximately 18% of the U.S. Armed Forces, suffer disproportionately higher rates of musculoskeletal injuries among active component service members. Using a retrospective study design, this study calculated incidence rates and rate ratios for acute hip fractures and hip stress fractures from January 1, 2018 through September 30, 2022 among female and male active component U.S. military members. Women who were younger than age 20 years, in recruit training, serving in the Army or Marine Corps, engaged in combat-related occupations, and with body mass indexes in the underweight or normal weight categories had the highest rates of both types of fractures. Women who had progressed beyond the recruit training phase had a higher female-to-male rate ratios of hip stress fractures than recruits. Despite an overall decline during the surveillance period, rates of acute hip fracture and hip stress fracture were higher among women than men. Changes in training and fitness policies may have contributed to the hip fracture rate declines among women. Continued efforts are needed to further reduce injuries among women.

**What are the new findings?:**

From January 2018 through September 2022, the recorded incidence of both acute and stress hip fractures among active component U.S military women declined while the female-to-male rate ratio remained elevated. The female-to-male rate ratio was alarmingly high for hip stress fractures in service women who had advanced beyond recruit training.

**What is the impact on readiness and force health protection?:**

This study includes data for hip fracture rates before and after several military branches implemented changes to training and physical fitness. These data could be used in concert with future studies of hip fractures to evaluate the efficacy of those changes in decreasing injury rates among military service women.

## 1 BACKGROUND

In December 2022 the U.S. Department of Defense (DOD) reinforced that women’s health equity remains a priority and that understanding the unique health needs of women is essential to addressing a variety of health conditions including musculoskeletal injuries (MSKIs). In 2022, injuries and musculoskeletal diseases were ranked first and third, respectively, for the attributable burden of illness in U.S. active component service members.^1^ MSKIs occur because of acute trauma or cumulative microtrauma from overuse to skeletal muscles, bones, tendons, joints, and ligaments. They can present as sprains, strains, fractures, dislocations, tendinitis, or bursitis and occur when mechanical energy is transferred to the tissue that exceeds the tissue’s tolerance.^2^

The most common sites for MSKIs among active component U.S. military populations include the lower extremities and spine.^3,4,5^ Among U.S. active duty Army service members in 2021, acute trauma accounted for 11% and microtrauma from overuse accounted for 70% of MSKIs.^2^ Women, who comprise 18% of the total U.S. military, tend to suffer higher rates of MSKIs than men.^2,5,6,7,8^ Furthermore, studies demonstrate that MSKIs differentially affect the hip regions in women.^5,6,7^ Hip fractures in particular account for a small percentage of all MSKIs in the military but can be disproportionately costly.^9^ They are defined as breaks in the upper portion of the femur bone and can occur acutely from traumatic forces or from the accumulation of microfractures from repetitive submaximal forces such as those that occur during strenuous physical training.^10^

Hip fractures result in increased health care provision, a significant amount of lost training or duty days, and high risk of medical separation from the military.^9,11^ The average cost of treating hip fractures in active duty soldiers in 2017 was estimated to be greater than $14,000 per case.^11^ Cases that required treatment in the inpatient setting exceeded the cost of treating other lower extremity fractures by approximately $5,000 to $10,000 per case.^11^ Soldiers who experienced hip fractures lost between 48 to 149 work days on average, depending on whether inpatient treatment was required.^11^ Furthermore, a different study found that 67% of active duty military members did not return to duty following surgical intervention for hip stress fracture (HSF).^12^ For Army recruits who experienced any type of lower extremity stress fracture during basic training, 33% of men and 42% of women were medically separated despite attempts at physical rehabilitation, which, on average, took greater than 70 days.^13^

Hip fractures pose a challenge for the military, not only in maintaining force readiness but for retention as well, for female service members in particular. Previous studies have focused on military recruits when assessing for HSFs and have not compared HSF rates in the different service components.^6,7,9,14,15^ No studies to date have examined rates of acute hip fracture (AHF) among military women and men. The aim of this study was to describe the incidence and potential risk factors of AHF and HSF among female active component U.S. service members compared to males. Associations between demographic factors and incidence of AHF and HSF were assessed, including age, race and ethnicity, branch of service, military occupation, and recruit status. In addition, the associations between body mass index (BMI) and both types of hip fractures were assessed.

## 2 METHODS

This study employed a retrospective cohort study design to determine female and male service members’ incidence rates of AHF and HSF. The surveillance period was January 1, 2018 through September 30, 2022. The population of interest included all individuals who served in the active component of the U.S. Army, Navy, Air Force, and Marine Corps. The study excluded U.S. Coast Guard members and reservists and personnel serving in the National Guard because the medical records for these populations were incomplete in the Defense Medical Surveillance System (DMSS) during the defined study period.

Data for this study pertaining to health encounters and demographic information were obtained from the DMSS.^17^ The International Classification of Diseases, 10th Revision, Clinical Modification (ICD-10-CM) seventh digit code was utilized to identify inpatient and outpatient AHF (S72 codes) and HSF (M84359A) encounters. A hip fracture was considered new if at least 60 days had passed since the last qualifying hip fracture encounter for an individual. For cases with inpatient and outpatient encounters on the same day, the inpatient encounter was selected for analysis.

BMI data came from the Military Entrance Processing Station (MEPS) and Periodic Health Assessment (PHA) databases within DMSS. For individuals with multiple weight records documented for a given calendar year, the last record of that year was used to calculate BMI. For individuals who had no weight records in a given year, their BMI category from the previous year was utilized.

Demographic factors included age, race and ethnicity, service branch, recruit status, and occupation. Age was categorized as: less than 20 years, 20-29 years, 30-39 years, and 40 years and older. For race and ethnicity, individuals were considered non-Hispanic White, non-Hispanic Black, Hispanic, or other/unknown. Service branch categories included the Army, Air Force, Navy, and Marine Corps. Occupational categories were obtained using Defense Enrollment Eligibility Reporting System DOD primary occupation codes for infantry, other combat-specific positions (which include artillery, armor, and combat engineer positions), motor transport, pilot and air crew, repair and engineering, communications and intelligence, health care, and other/unknown (**Table 1**). The other/unknown category included new recruits.

Incidence rates were calculated per 10,000 person-years (p-yrs). Female and male service members were analyzed separately. Incidence rate ratios (RRs) and their corresponding 95% confidence intervals (CIs) were calculated to compare injury rates between women and men.^16^ BMI was calculated as body weight (lb.)/height^2^ (in^2^) x 703). BMI was categorized as underweight (<18.5), healthy weight (18.5-24.9), overweight (25.0-29.9), and obese (>=30).

## 3 RESULTS

From January 1, 2018 through September 30, 2022, 2,886 incident cases of hip fractures occurred over a total of 6,220,311 p-yrs. Of the total cases, 44% were AHF (356 in females and 924 in males) and 56% were HSF (802 in females and 804 in males). **Table 2** summarizes incidence rates of AHF and HSF among women and men by demographic factors and BMI. For both AHF and HSF, higher incidence rates occurred in female compared to male service members.

Female service members under age 20 years experienced AHF and HSF at rates 4.7 times (95% CI: 3.5, 6.2) and 6.8 times (95% CI: 5.7, 8.1) higher than males in the same age group, respectively. Women between 20 and 29 years old had 1.7 times (95% CI: 1.5, 2.0) and 3.8 times (95% CI: 3.3, 4.3) higher rates of AHF and HSF than men in the same age group, respectively. Higher rates of hip fractures were seen in women compared to men in all racial and ethnic groups. Female Hispanic service members in particular experienced HSF at a rate 6.2 times (95% CI: 5.0, 7.7) higher than males.

Female service members serving in the Army and Marine Corps sustained AHF at rates 2.2 times (95% CI: 1.9, 2.6) and 4.6 times (95% CI: 3.6, 6.1) higher, respectively, than those in males. The RR of HSF between female and male service members were greater than 4.0 in all 4 services, with the highest RR between female and male Marines at 14.4 (95% CI: 11.7, 17.8). Female recruits sustained higher rates of both AHF and HSF than male recruits (RR 3.1; 95% CI: 2.3, 4.0 and RR 3.5; 95% CI: 3.0, 4.1), respectively. The female-to-male RR for HSF was larger for non-recruits than recruits (6.1; 95% CI: 5.3, 6.9 and 3.5; 95% CI: 3.0, 4.1), respectively, however.

The female-to-male RR for AHF was highest for combat-specific occupations (RR 2.9, 95% CI: 1.8, 4.8). The female-to-male RR for HSF was highest for combat-specific (RR 6.6, 95% CI: 4.4, 9.9) and repair/engineer (RR 6.6, 95% CI: 5.2, 8.4) occupations. Few women in this cohort identified as having infantry or pilot/air crew occupations.

At a maximum, BMI measurements were obtained 2 years from the HSF date. Of the 1,369 HSF cases that had a BMI measurement within 2 years, the median time between fracture and BMI measurement was 82 days and the mean was 108 days. Of the 1,024 AHF cases that had a BMI measurement within 2 years, the median time between AHF and BMI measurement was 119 days and the mean was 163 days. There was an inverse relationship between BMI and hip fracture among both sexes.

**Figures1a** and **1b** show an overall decline in the incidence rates of AHF by 48% and HSF by 63% in women, respectively. Despite this downward trend among cases of hip fractures, women continued to experience higher rates of hip fractures than men during each year of the surveillance period.

## 4 DISCUSSION

The overall incidence of AHF and HSF in women in the active component U.S. military in this study declined between 2018 and 2022. The decline in hip fractures may be in part due to the COVID-19 pandemic, which affected physical training activities due to mandates to stay at home and maintain social distancing. Another possible explanation for this decline may be due service-specific changes such as those made by the Army and Marine Corps. In 2019, the Marine Corps started training women and men in sex-integrated recruit units, which was associated with fewer injuries among individuals.^18^ In 2020, the Army implemented its Holistic Health and Fitness program and a new physical fitness test to reduce MSKIs by promoting whole-body strength-training.^19^

A prior study that assessed HSF from 2009 to 2012 reported incidence rates of 16.8 and 2.9 per 10,000 p-yrs for active duty women and men, respectively.^20^ Comparatively, the incidence rates in our study were lower: 7.6 and 1.6 per 10,000 p-yrs for women and men, respectively. This downward trend may reflect the military’s increased investments in funding research and programs to prevent injuries while improving performance and rehabilitation after injuries.^21,22^

Despite the overall decline, the rates of hip fractures in this study were higher for women for each year of the surveillance period when compared to men. Differences between women and men for anatomical, biomechanical, physiological, and fitness factors have been cited as contributing to higher rates of injuries in military women.^5,6,7,15^ Women tend to have wider pelvises, longer femora, and narrower bones, on average, with thinner cortices than men.^15^ Physiological factors related to irregular menstrual cycles and iron deficiency have also been found to increase risk of stress fractures in military women.^6^

In this study, age younger than 20 years was associated with higher risk of both AHF and HSF in women, consistent with previous data.^20,23^ One study in Army recruits reported that age older than 20 years conferred higher risk of stress fractures overall.^24^ That study did not, however, stratify the data by site of fracture and included stress fractures of the lower leg and foot.^24^

Unexpectedly, while the rate of HSF in women who had progressed beyond the recruit period was lower overall than those going through recruit training, the female-to-male RR was higher for women outside the recruit training phase (6.1 vs. 3.5 per 10,000 p-yrs). These findings highlight that women are at higher risk than men for life- and career-changing injuries throughout their military service. Many studies have previously focused on injury rates between female and male recruits.^7,9,14,25^ No studies, however, have compared rates of either type of hip fractures in women in recruit training versus operational units. Sustaining hip fractures may result in permanent physical restrictions, which affect service members’ opportunities for progression and retention.^6^ MSKIs in general were associated with higher rates of discharge in women when compared to men.^6^

Army or Marine Corps service and a combat-related occupation also placed women in this study population at increased risk of AHF in comparison to men. Marine Corps and a combat-related or repair/engineer occupations placed women at higher risk of HSF than men. There were few women in the infantry-specific occupations, and as a result, no AHFs and few HSFs were seen in women during the surveillance period. These findings are consistent with published fndings.^5,20,23^ For military women, researchers found that marching with a heavy load and running long distances resulted in a higher proportion of MSKIs when compared to men.^5,6^ Lower levels of strength and aerobic fitness upon entering basic training were also associated with higher risk of injuries, specifically in female recruits.^6,7,14^

While this study was unable to assess for any associations between level of fitness and risk of hip fracture, these results show an inverse relationship between BMI and incidence of hip fractures among women and men. Higher rates of hip fractures were seen among service members in the underweight and normal weight categories. These findings are counterintuitive, given the evidence that higher BMI was shown to be associated with higher rates of MSKIs.^26^ Women in all BMI categories sustained higher rates of hip fractures than men, except for women in the obese category, in relation to AHF. Previous studies have shown an association between low BMI and higher risk of stress fractures among military populations.^6,24,27^ This study did not adjust for confounding nor modifying factors, and comparisons were made based on crude rates.

This study has other limitations. BMI data obtained from MEPS and PHAs were self-reported. Furthermore, this study was unable to quantify the long-term impact of hip fractures and whether that differed for men versus women. Rates of medical discharge, numbers of lost duty days, durations of physical restriction, and permanent disabilities resulting from hip fractures were not assessed. No studies to date, however, had assessed for risk of AHF in relation to BMI, making our findings unique. Future studies are needed to identify modifiable factors such as nutrition or training techniques that can mitigate the risk for devastating fractures in military women, as well as track duration of rehabilitation and rate of medical discharges after hip fractures.

## Figures and Tables

**Table 1:** DEERS DOD Codes Used to Identify Military Occupations

Occupation	Code(s) that begin with:
Infantry	1010
Other, combat-specific	101, 102, 103, 104, 143, 220500
Motor transport	106, 181, 280300
Pilot/air crew	105, 220100, 220200, 220300, 220400
Repair/engineering	11, 16, 17, 24
Communications/intelligence	12, 15, 23, 27
Health care	13, 26
Other/unknown	Other

Abbreviations: DEERS, Defense Enrollment Eligibility Reporting System; DOD, Department of Defense.

**Table 2:** Incidence of Acute Hip Fractures and Hip Stress Fractures Among Active Component U.S. Service Members by Demographic Characteristics, 2018–2022

Acute Hip Fractures							
	Female		Male		Female/Male		
	No.	Rate^a^	No.	Rate^a^	RR	95% LL	95% UL
Total	356	3.4	924	1.8	1.9	1.7	2.1
Age group, y							
$lt;20	104	12.4	96	2.7	4.7	3.5	6.2
20–29	206	3.4	548	1.9	1.7	1.5	2.0
30–39	35	1.3	205	1.4	0.9	0.6	1.3
40+	11	1.3	75	1.5	0.9	0.5	1.7
Race and ethnicity							
White, non-Hispanic	158	3.6	603	2.0	1.8	1.5	2.1
Black, non-Hispanic	66	2.6	130	1.8	1.5	1.1	2.0
Hispanic	90	4.3	130	1.5	2.8	2.2	3.7
Other	42	2.8	61	1.1	2.6	1.8	3.9
Branch of service							
Army	195	5.7	484	2.6	2.2	1.9	2.6
Navy	42	1.3	140	1.1	1.2	0.8	1.7
Air Force	42	1.3	131	1.1	1.2	0.9	1.7
Marine Corps	77	10.0	169	2.2	4.6	3.6	6.1
Occupation							
Infantry	0	0.0	90	2.3	0.0	--	--
Other, combat-specific	18	6.2	94	2.1	2.9	1.8	4.8
Motor transport	17	5.1	38	2.5	2.1	1.2	3.7
Pilot/air crew	3	1.8	22	1.1	1.7	--	--
Repair/engineering	53	2.5	239	1.5	1.7	1.3	2.3
Communications/intelligence	87	2.6	144	1.4	1.8	1.4	2.3
Health care	60	3.1	55	1.6	1.9	1.3	2.7
Other/unknown	118	5.0	242	2.4	2.1	1.7	2.6
Recruit basic training							
Yes	84	37.5	128	12.3	3.1	2.3	4.0
No	272	2.6	796	1.6	1.7	1.5	1.9
Body mass index							
Obese (>=30)	12	1.3	87	1.3	1.0	0.6	1.9
Overweight (25<=30)	83	2.8	317	1.6	1.8	1.4	2.3
Normal weight (18.5<=25)	178	4.8	311	2.4	2.0	1.7	2.4
Underweight (<18.5)	11	9.2	9	3.1	2.9	1.2	7.1
Unknown	72	2.5	200	1.7	1.5	1.1	1.9

**Hip Stress Fractures**							
	**Female**		**Male**		**Female/Male**		
	**No.**	**Rate^a^**	**No.**	**Rate^a^**	**RR**	**95% LL**	**95% UL**
Total	802	7.6	804	1.6	4.9	4.4	5.4
Age group, y							
<20	328	39.0	207	5.7	6.8	5.7	8.1
20–29	416	6.8	509	1.8	3.8	3.3	4.3
30–39	52	1.9	79	0.5	3.5	2.4	4.9
40+	6	0.7	9	0.2	4.2	1.5	11.9
Race and ethnicity							
White, non-Hispanic	344	7.8	502	1.7	4.6	4.0	5.3
Black, non-Hispanic	176	6.8	105	1.4	4.8	3.8	6.1
Hispanic	211	10.2	139	1.6	6.2	5.0	7.7
Other	71	4.8	58	1.0	4.7	3.4	6.7
Branch of service							
Army	528	15.5	607	3.2	4.8	4.3	5.4
Navy	22	0.7	20	0.2	4.3	2.4	7.9
Air Force	36	1.1	24	0.2	5.6	3.3	9.4
Marine Corps	216	28.2	153	2.0	14.4	11.7	17.8
Occupation							
Infantry	3	17.1	72	1.9	9.1	--	--
Other, combat-specific	34	11.8	78	1.8	6.6	4.4	9.9
Motor transport	20	6.0	24	1.6	3.8	2.1	6.9
Pilot/air crew	3	1.8	1	0.1	36.2	--	--
Repair/engineering	118	5.6	139	0.9	6.6	5.2	8.4
Communications/intelligence	201	6.0	136	1.4	4.4	3.5	5.5
Health care	96	4.9	38	1.1	4.4	3.0	6.4
Other/unknown	327	13.9	316	3.1	4.4	3.8	5.2
Recruit basic training							
Yes	300	134.1	398	38.3	3.5	3.0	4.1
No	502	4.9	406	0.8	6.1	5.3	6.9
Body mass index							
Obese (>=30)	11	1.2	49	0.7	1.7	0.9	3.3
Overweight (25<=30)	195	6.6	245	1.2	5.4	4.4	6.5
Normal weight (18.5<=25)	464	12.5	356	2.8	4.5	3.9	5.2
Underweight (<18.5)	29	24.3	18	6.3	3.9	2.2	7.0
Unknown	103	3.6	136	1.2	3.1	2.4	4.0

Abbreviations: No., number; y, years; RR, rate ratio; LL, lower limit; UL, upper limit.

Note: Confidence interval estimates are unstable due to small group sizes.

^a^Rate per 10,000 person-years.

**Figure 1a:**
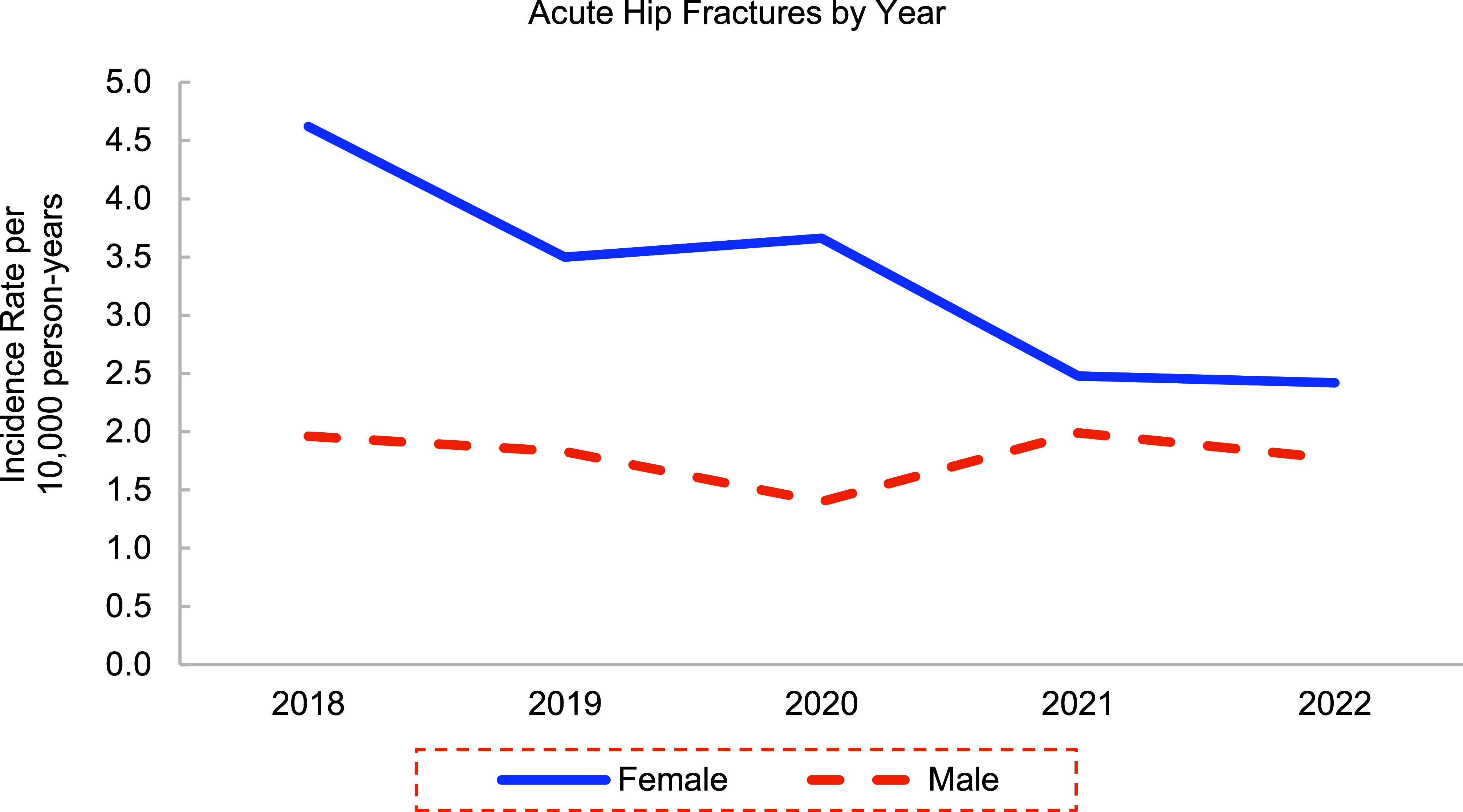
Incidence Rates of Acute Hip Fractures Among Active Component U.S. Service Members by Year, 2018–2022

**Figure 1b:**
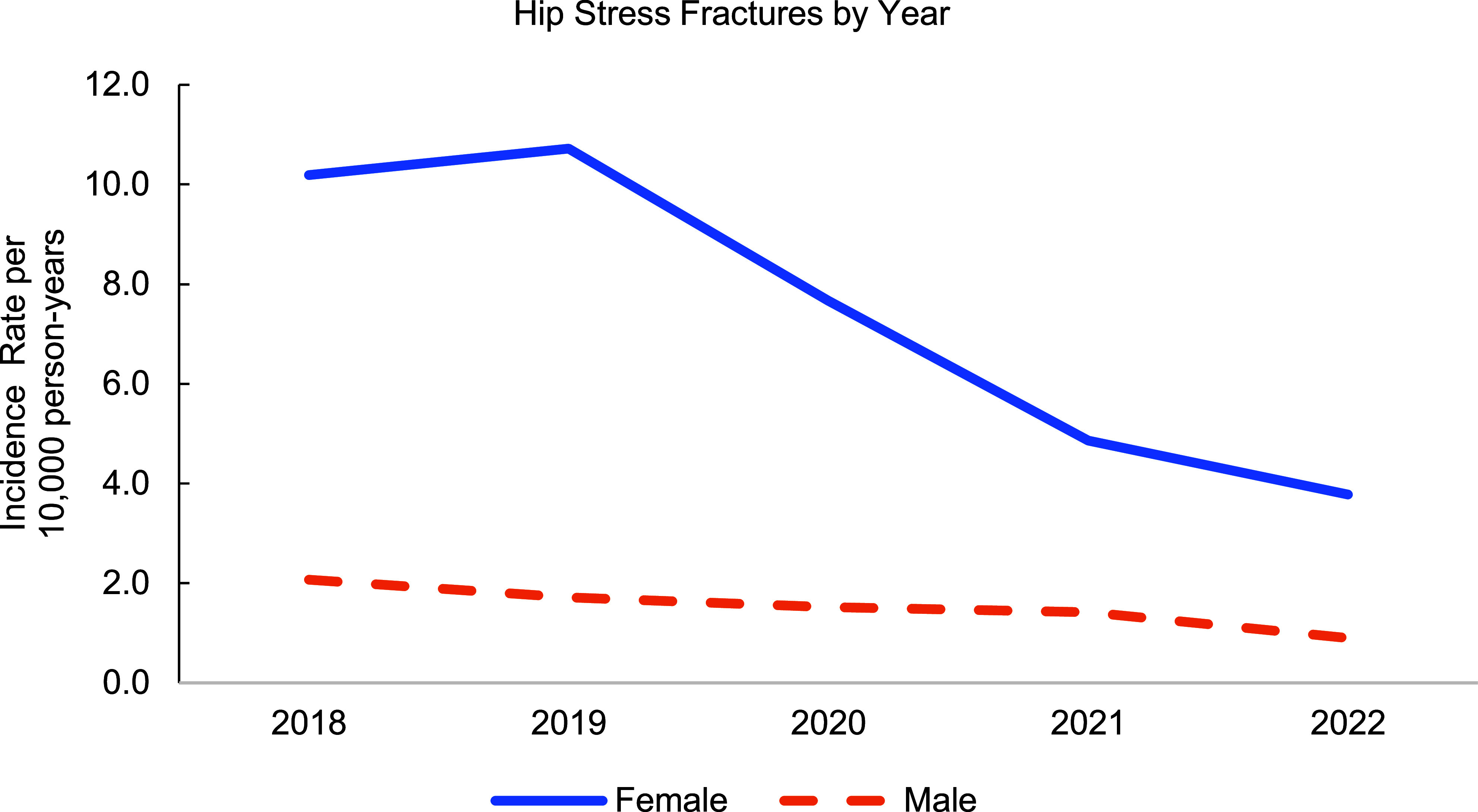
Incidence Rates of Hip Stress Fractures Among Active Component U.S. Service Members by Year, 2018–2022

## AUTHOR AFFILIATIONS

Fort Leonard Wood Department of Public Health, MO: CPT Vu; Armed Forces Health Surveillance Division, Silver Spring, MD: Dr. Stahlman, CAPT Wells
